# Insights into Interactions between Interleukin-6 and Dendritic Polyglycerols

**DOI:** 10.3390/ijms22052415

**Published:** 2021-02-28

**Authors:** Željka Sanader Maršić, Dušica Maysinger, Vlasta Bonačić-Kouteckỳ

**Affiliations:** 1 Faculty of Science, University of Split, Ruđera Boškovića 33, HR-21000 Split, Croatia; 2 Center of Excellence for Science and Technology-Integration of Mediterranean Region (STIM) at Interdisciplinary Center for Advanced Sciences and Technology (ICAST), University of Split, Meštrovićevo šetalište 45, HR-21000 Split, Croatia; vbk@cms.hu-berlin.de; 3 Department of Pharmacology & Therapeutics, McGill University, Montréal, QC H3G 1Y6, Canada; 4 Department of Chemistry, Humboldt Universitat zu Berlin, Brook-Taylor-Strasse 2, 12489 Berlin, Germany

**Keywords:** interleukin-6, inflammation, heparin, dendritic polyglycerols, molecular docking, anti-inflammatory nanostructures, protein-dendrimer interaction

## Abstract

Interleukin-6 (IL-6) is involved in physiological and pathological processes. Different pharmacological agents have been developed to block IL-6 deleterious effects and to recover homeostatic IL-6 signaling. One of the proposed nanostructures in pre-clinical investigations which reduced IL-6 concentrations is polyglycerol dendrimer, a nano-structure with multiple sulfate groups. The aim of the present study was to uncover the type of binding between critical positions in the human IL-6 structure available for binding dPGS and compare it with heparin sulfate binding. We studied these interactions by performing docking simulations of dPGS and heparins with human IL-6 using AutoDock Vina. These molecular docking analyses indicate that the two ligands have comparable affinities for the positively charged positions on the surface of IL-6. All-atom molecular dynamics simulations (MD) employing Gromacs were used to explore the binding sites and binding strengths. Results suggest two major binding sites and show that the strengths of binding are similar for heparin and dPGS (−5.5–6.4 kcal/ mol). dPGS or its analogs could be used in the therapeutic intervention in sepsis and inflammatory disorders to reduce unbound IL-6 in the plasma or tissues and its binding to the receptors. We propose that analogs of dPGS could specifically block IL-6 binding in the desired signaling mode and would be valuable new probes to establish optimized therapeutic intervention in inflammation.

## 1. Introduction

Interleukin-6 (IL-6) has been extensively investigated because it is involved in physiological and pathological processes. Many therapeutic interventions have been applied to maintain physiological signaling and reduce or abolish deleterious ones. IL-6 exerts its effects via three signaling modes: classical, trans-signaling, and trans-presentation [1]. Clinical interventions and preclinical studies in experimental animals have provided results suggesting that the selection of patients and animal models is of critical importance in the selection of therapeutic intervention [1]. IL-6 concentrations under physiological conditions are very low (1–5 pg/mL) but in inflammation and sepsis they can reach high nanomolar range. IL-6 in high concentrations can cause serious neurological impairments. A balance between concentrations, extent, mode of binding to IL6 receptors, and signaling pathways determines the outcome of these events to be beneficial or damaging [2]. Thus, IL-6 regulation needs to be controlled and finely tuned to maintain homeostasis. The aim of our studies was to show if some dendrimer nanostructures with sulfate groups can bind to the hypothesized positively charged sites in IL-6 structure and if so, what are the properties and binding strengths at these sites.

Human interleukin-6 (hIL-6) is a soluble cytokine, consisting of 185 residues [3]. Its structure has been resolved and it consists of four alpha-helices with loop regions of variable length (forming a classical four-helix bundle), with the fifth helix located in the loop between helices C and D [4,5]. It is produced in response to injuries and infections stimulating the immune reactions [6]. In clinical practice, IL-6 is associated with cardiovascular disease, cancer, osteoporosis, and can be used as a biomarker in sepsis [7]. It is involved in various neurological diseases, because it can be produced by activated glial cells [8]. IL-6 is abundant in plasma and tissues in several chronic diseases, like rheumatoid arthritis [9,10], Castleman’s disease [11], and brain tumors such as glioblastoma etc. For this reason, finding the modes of regulating IL-6 expression, protein abundance, and signal transduction is critical to help prevent and reduce severity and duration of pathological state implicating excessively high IL-6 concentrations.

Dendritic polyglycerols are glycerol-based hyperbranched structures with huge variety of sizes (at the nanoscale) and functional end groups [12,13] which enable them to interact with different biological partners [14]. Dendritic polyglycerol sulfates (dPGS) are polyglycerols with sulphate groups; they can prevent hyperactivation of glial cells and attenuate inflammatory responses [15,16], mostly due to their heparin-mimicking properties [17,18]. We reported experimental data showing concentration and time-dependent effects of dPGS in vitro and in vivo on rodent glial cells [15,19,20].

Heparin is a polymer from the glycosaminoglycan family which is built from disaccharide units of uronic acid (either d-glucuronic acid or l-iduronic acid) and d-glucosamine part with varying number of sulfate groups [21,22]. The most common disaccharide subunit consists of a 2-O-sulfated iduronic acid and 6-O-sulfated, N-sulfated glucosamine [23].

Due to its interaction with numerous proteins, heparin plays an important role in many biological processes [24] such as enhancing the activity of basic fibroblast growth factor [25]. On the other hand, its influence on cytokines, especially, interleukins is often inhibitory [26,27,28,29].

It is already known that IL-6 binds to sulfated glucosaminoglycans (GAGs) [30]; this can keep this small protein close to the injured area preventing its diffusion to healthy parts of the host. More specifically, heparin binding sites in proteins are usually composed of four to six basic amino acids, Arg or Lys [24], which can be clustered at one site, or separated by approximately 20 Å which corresponds to the positioning of sulfate groups along the GAGs. Figure S1 in the Supporting information presents structure of the heparin (pdb: 1HPN [31]) illustrating the distance between the sulfate groups. Such positively charged cluster of amino acids is present in the hIL-6, and it includes amino acids: Arg41, Lys42, Arg169, and Lys172 at one site and Lys28, Arg31, Arg180 and Arg183 at site very close to the first group [32]. dPGSs are heparin analogues which makes them plausible candidates for IL-6 binding. Indeed, it has been shown that treatment with dPGS can reduce IL-6 release from microglia [15] and attenuate reactivity of astrocytes due to the secreted factors from hyperactive microglia [19].

The structure of dPGS in solution has been investigated and described [33] as well as the process of the binding of the dPGS to proteins. It was shown that this binding is governed by the counterions release from the surface of the dPGS [34]. This study showed weak dependence of the binding free energy on the size (generation) of the dPGS. MD simulations illustrated the importance of the counterions release contribution to the binding free energy and evaluated the electrostatic screening part of it which is found to be smaller than the counterions release part. In the present work, we study the interaction of heparin and dPGS with hIL-6 upon counterions release as well as their binding properties, using theoretical modeling to determine binding affinities of both ligands toward hIL-6 and to examine the different types of interactions between them. We addressed the questions: (1) Do dPGS and heparin bind to the same sites? and (2) Are the bonds between dPGS and IL-6 comparable with heparin—IL-6? For this purpose, molecular docking and molecular dynamics (MD) simulations were applied to determine the noncovalent interactions between a protein and a ligand (usually small molecule). Molecular docking is a very useful technique in the field of drug design and is used for virtual screening. It facilitates fast in silico examination of databases of potential drugs to identify those which are likely to bind to a target protein. It is used for prediction of a binding pose, that is the position and orientation of a ligand relative to a protein which can serve to design of a more effective and selective analogs. All-atom molecular dynamics (MD) simulations provide the opportunity to investigate a molecular system at the atomic level and they are usually compatible to experimental data (X-ray crystallography, nuclear magnetic resonance, electron paramagnetic resonance…). In MD simulations, equations from Newton’s Laws of motion are solved to predict the path of atoms over time revealing the conformational changes of a protein or its interaction with different ligands.

We investigated the possibility of dPGS/heparin substitution and inhibition of the heparin-binding protein (hIL-6) with dPGS. Molecular docking can show if the binding sites of heparin and dPGS to the proteins are the same and can be used to describe the nature of their binding (e.g., ionic, hydrophobic, etc). In addition, MD simulations were used to further analyze the poses obtained by docking. These calculations confirmed the docking results giving us time evolution of the complexes. Our present study was stimulated by numerous experimental data for the IL-6, interplay between glial cells in humans and rodents in inflammation involving IL-6 and dPGS as a nano-modulator in inflammation. Comparison of a human IL6 sequence (pdb: 1IL6) and a rodent one (pdb: 2L3Y) reveals the homology of the described groups of the positively charged amino acids. Namely, Arg169 in human IL6 is exchanged with another positively charged amino acid (Lys) while Arg41, Lys42, Arg180, and Arg183 are homologous. Considering the similarities between the human and rodent IL-6 positively charged domains, the findings from the current study suggest that dPGS structural analogs could be designed to exert superior modulatory effects on glia by stronger binding to human and rodent proteins with similar domains.

## 2. Results

### 2.1. Molecular Modeling Aspects

**Figure 1:**
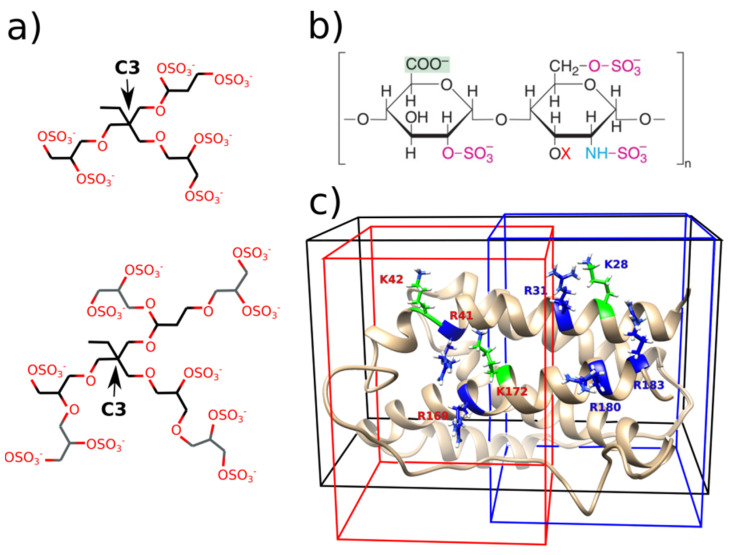
(**a**) Sketch of the first and the second generation of the dPGS. Gray colored carbons are additional branches that mark the second generation. Indicated C3 atom is used in the analysis of the MD simulations; (**b**) Structure of the heparin disaccharide unit, red X marks the variably sulphated position, which means that X=H or SO_3_^−^. (**c**) Structure of hIL-6 with the highlighted amino acids used to define the binding sites and the corresponding docking boxes. Red labels and red colored box for RKRK site, and blue labels, blue colored box for KRRR site. Black box includes both binding sites RKRK&KRRR. Structures are not up to scale.

We studied the interaction between the human interleukin-6 (hIL-6) and ligands: (I) dendritic polyglycerol sulfate of the first and second generation and (II) heparins of different lengths and number of SO_3_^−^ groups. Therefore the strategy for docking procedure included two types of disaccharide unit of heparin: one with three SO_3_^−^ groups, and with one OH group (at the X position marked on Figure 1b), while the other one has four SO_3_^−^ groups (structure sulphated at the X position on Figure 1b). A series of docking simulations of ligands to hIL-6 with AutoDock Vina was preformed to evaluate the binding sites and binding strength of the ligands. Since the ligands are negatively charged the possible binding sites are defined by the positively charged amino acids [32] as described in Section 4, Materials and Methods.

We used this simple qualitative molecular modeling to determine amino acids involved in interactions with dPGS and heparin as well as to identify their position and to calculate strengths of their binding.

Furthermore, we used MD simulations to test the stability of the complexes obtained by docking.

### 2.2. dPGS Docking to hIL-6

**Figure 2:**
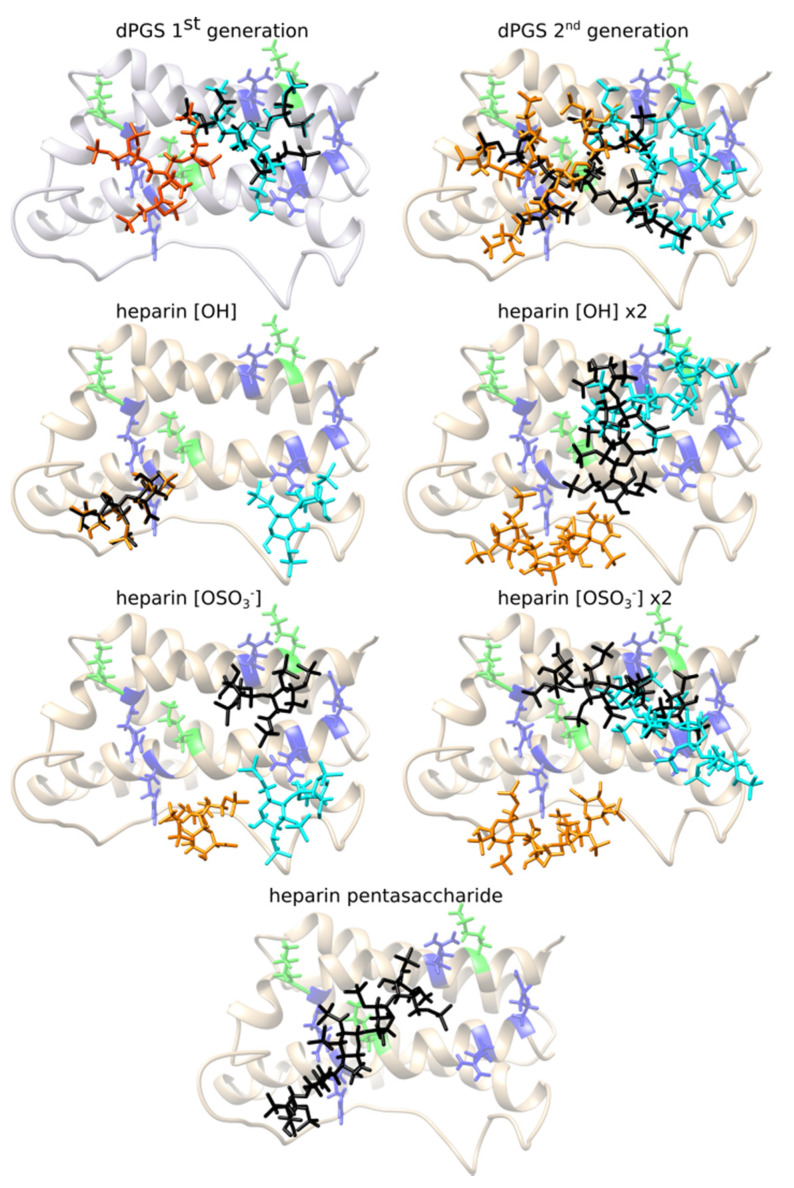
The most favorable binding positions for different ligands and binding sites of IL-6. Lysine form the docking sites is colored green, arginine is colored blue. Orange structure represents the corresponding pose obtained in the RKRK docking box; cyan pose obtained in the KRRR docking box, and black is the pose obtained in the docking box (iii) RKRK&KRRR which surrounds both binding sites.

Due to the dPGS branched structure, a large number of structures can be formed by rotation of C-C and ester bonds. Our strategy here includes modeling of dPGS of the first generation with 6 -OSO_3_^−^ groups and second generation with 10 -OSO_3_^−^ groups (compare Figure 1a). In the latter case, dPGS has 53 active torsions which makes it the most difficult ligand to dock. For this reason, docking calculations were repeated 100 times, and the docking pose was selected according to the largest binding affinity and its appearance as the preferable binding position in the most runs. Similar procedure was applied for the other ligands, but the difference is that the number of the docking calculations needed to achieve convergence and to find the binding position is considerably smaller. Binding affinities of the ligands to different binding sites of hIL-6 are given in the Table 1. The structures corresponding to the most favorable docking positions of ligands are shown in Figure 2.

Two groups of four positively charged amino acids (R41, K42, R169, K172 and K28, R31, R180, R183) form two quadrilateral shapes on the surface of the two α-helices of the IL-6: α1-helix and α6-helix (compare Figure S2). Area of these shapes fits the size of the dPGS of the first generation which can bind either to amino acids from the RKRK or from the KRRR docking box forming interactions with 12 amino acids in average. Its binding position in the largest docking box (iii) RKRK&KRRR still involves mostly amino acids from the one binding site (the KRRR site). Analysis of the 20 different docking simulations with the lowest docking scores in the box (iii) RKRK&KRRR reveals that all 20 lowest energy poses are in the KRRR site. Binding energies are in range −6.0 kcal/mol to −5.7 kcal/mol.

dPGS of the second generation has 53 active torsions and its structure is very flexible. Its size exceeds the area of these quadrilateral shapes and it interacts with ~20 amino acids surrounding the R41, K42, R169, K172 and K28, R31, R180, R183. Due to its high flexibility it can interact with amino acids from only one box at the time. But, its favorable binding position in the box iii) RKRK&KRRR includes combination of amino acids from both binding sites (compare Figure 2 and Figure S2). The docking pose in this case stretches along the α1-helix and α6-helix and, assuming that its structure is spherical, its diameter is ~24 Å.

### 2.3. Heparin Docking to hIL-6

Heparin is polymer consisting of a repeating disaccharide unit which can differ in the number of SO_3_^−^ groups (compare the Figure 1b). The aim was to investigate the influence of these two different types of units (with OH or SO_3_^−^ group) on the binding affinities. In addition, we elongated structure of heparin by binding two disaccharide units, and at the end we docked heparin pentasaccharide with combination of sulfated disaccharide units.

The disaccharide heparin is long approximately 13 Å and it interacts with amino acids from one binding site at the time. Its interactions include 11 and 15 partner amino acids from hIL-6 for disaccharide heparin [OH] and disaccharide heparin [OSO_3_^−^] respectively. Elongated tetrasaccharide heparin is ~17 Å long and it can fit the RKRK and KRRR box separately. Its docking pose in the box (iii) RKRK&KRRR includes amino acids from both sites. We analyzed the lowest energy structures obtained in the 20 different docking simulations in the box (iii) RKRK&KRRR. Binding scores for the analyzed structures are in range from −6.5 kcal/mol to −6.3 kcal/mol. Among them 15 poses are in the RKRK part of the box (iii), while five of them are in the KRRR part. This shows in the box (iii) the poses in the RKRK site occur with greater frequency, and that the docking scores obtained are very similar for both sites. Slight difference in the docking scores for the OH and SO_3_^−^ heparin disaccharide and tetrasacharide subunits is not sufficiently significant to indicate any difference. The Arpeggio analysis reveals that the heparins with OH and SO_3_^−^ group interact mostly with the similar amino acids, e.g., both tetrasaccharides in the RKRK site interact with R59, L63, K67, M68, H165, R169, S170, E173, F174, while in the KRRR site amino acid partners include: R31, Q176, R183. In the case of the disaccharide unit of heparin, binding occurs to slightly different places in the RKRK box which makes them interact with a different amino acids (see Figure 2), but the KRRR box reveals again the same interactions to residues: C74, Q76, S177, R180, Q184 (compare Figure 4).

The heparin pentasaccharide used in this analysis is a step towards a realistically elongated structure of heparin, with variably sulphated X position (Figure 1b). Structure studied here has three X positions with following functional groups: OH group, OSO_3_^−^ and OH group. The length of the heparin pentasaccharide (~25 Å) prevents it from binding to the separated docking boxes of the RKRK and KRRR site (length 22 Å), and it binds to the amino acids from the both sites simultaneously in the box iii) RKRK&KRRR (length 42 Å). Its extended structure stretches along the surface of the hIL-6 from the amino acids belonging to the RKRK binding site to the amino acids at the beginning of the KRRR site (Figure 2). The rest of the amino acids in the KRRR site are not interacting with the heparin pentasaccharide which indicates that the even longer heparin could be positioned there.

We found that the presented docking scores: in the range of −5.7 kcal/mol to −6.6 kcal/mol for disaccharide heparin; from −5.5 kcal/mol to −6.5 kcal/mol for tetrasaccharide heparin; and −6.4 kcal/mol for pentasaccharide heparin, are all very similar and we did not find any preference among the poses.

**Figure 3:**
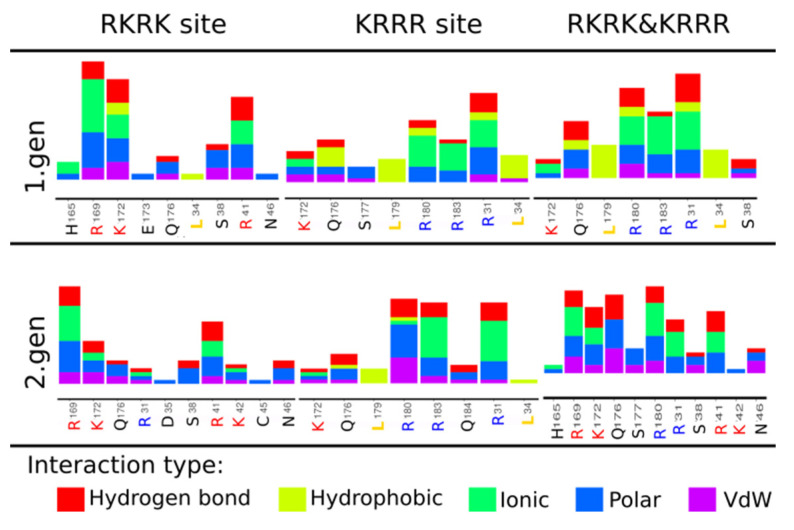
Arpeggio analysis for the dPGS to hIL-6.

**Figure 4:**
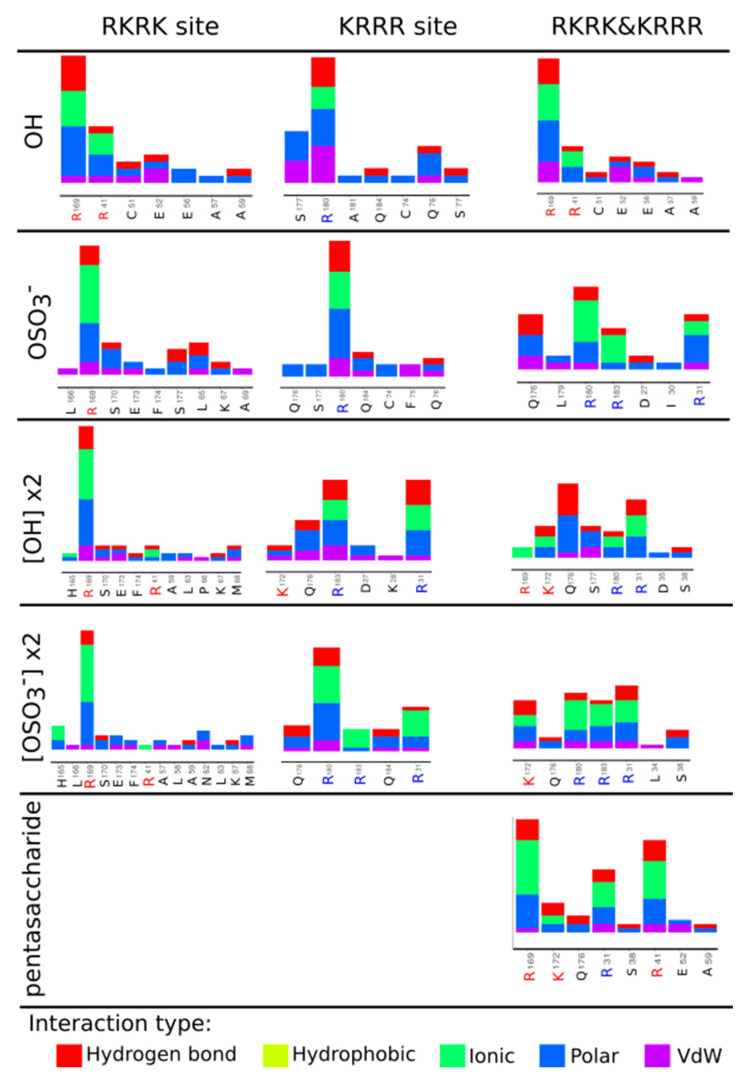
Arpeggio analysis for the different heparin structures to IL-6.

In addition, analysis of the obtained binding positions by the type of interactions between the ligands and hIL-6 was performed using Arpeggio and it is summarized in Figure 3 (dPGS) and Figure 4 (heparin). The number of residues forming interactions increases as the structures of the dPGS and heparin grow longer. Arpeggio analysis confirmed the existence of ionic interactions between negatively charged SO_3_^−^ groups from dPGS and positively charged side chains of amino acids at the RKRK and KRRR sites. Additionally, identification of other types of the interactions (such as hydrogen bonds, hydrophobic, polar, and Van der Waals interactions) with the other residues in vicinity of the ionic ones has been successfully made. Polar interactions and hydrogen bonds are present for all the ligands and they contribute to the binding affinities. Residues forming these interactions are very similar for dPGS of the first and the second generation and include E173, Q176, and S38 (see Figure 3). As far as for heparin these residues differ slightly between disaccharide heparin and longer forms because of the difference in length of disaccharide (14 Å) and pentasaccharide (25 Å) heparin. Residues such as C51 and E52 form the polar interactions and hydrogen bonds in disaccharide heparin, while longer forms include interactions with residues E173, Q176, E52, and S38 (see Figure 4).

Comparison of the binding sites and the interaction types between the hIL-6 and investigated ligands indicates that heparin can be replaced by dPGS. Due to the specific elongated structure of heparin with an almost equal distance between negatively charged sulfate groups, binding sites on IL-6 include similarly spaced positively charged amino acids [32]. Lack of such additional binding sites suggests that heparin binds in the RKRK&KRRR area in agreement with the molecular docking analysis. The size of the dPGS of the second generation, the number of negatively charged sulfate groups, and similar binding scores to heparin, all indicate that the favorable binding site would be the RKRK&KRRR site. However, smaller dPGS of the first generation might find additional binding spots on the surface of IL-6. Docking simulations are adequate for describing binding after the counterions release which can be addressed using MD simulations. Moreover, docking provides information about binding sites, which is crucial for the study of dPGS in replacing heparin.

### 2.4. Molecular Dynamics Simulations of the dPGS and hIL-6

Interactions between hIL-6 and dPGS were further analyzed using MD simulations as described in Section 4. Materials and Methods. Poses obtained through docking and described in Section 2.2 were starting structure for MD simulations. We collected a total of 12 MD simulations for six poses we obtained from docking for dPGS of the first and the second generation. These 12 MDs include: (i) 100 ns MDs for each pose from the docking (six in total), and (ii) additional set of MDs starting from the same pose, but with different initial velocities (six MDs). These second MDs are necessary to ensure the reproducibility of the results and they are expected to show the same behavior as the first set of MDs. Moreover, to ensure stability on a longer timescale, we have elongated six MDs to 200 ns.

The first analysis we preformed was an investigation of the minimal distance between the protein and its periodic image to eliminate the possibility of any artifacts from the interactions of the protein with itself. For all 12 MDs, these distances are adequate, and in all cases greater than 2.4 nm (see Figure S3).

**Figure 5:**
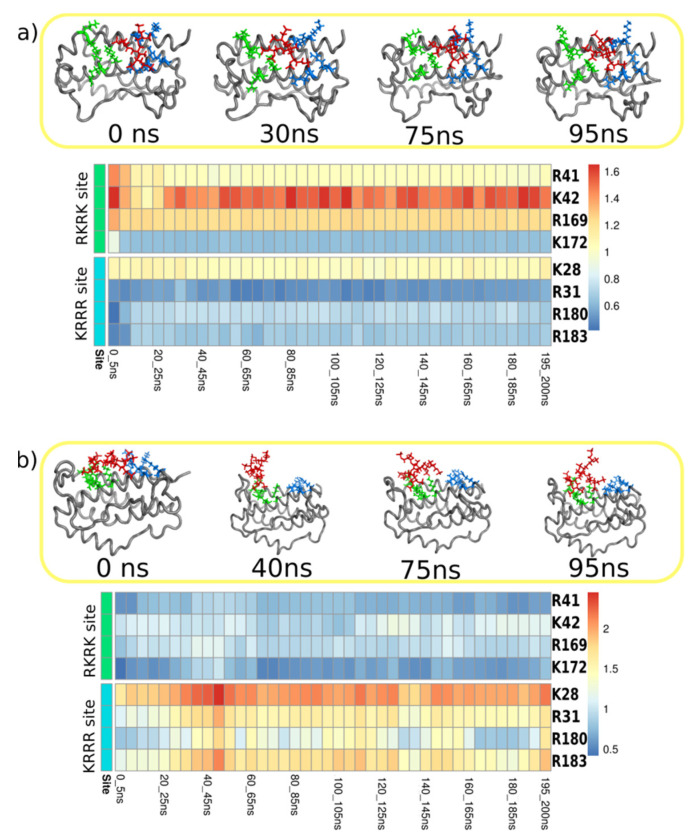
Analysis of the MD simulations for two docked poses into RKRK&KRRR box: (**a**) dPGS of the first generation; and (**b**) dPGS of the second generation. Upper parts show snapshots from the MD, with IL6 colored grey, dPGS colored red, amino acids from RKRK site are green and amino acids from KRRR skyblue. Heat maps present analysis of the distances between central C3 atom of the dPGS and positively charged amino acids from RKRK (R41, K42, R169, K172) and KRRR (K28, R31, R180, R183) site during 200-ns MD. Heat map represents distances from 0.4 to 1.6 nm where blue color indicated shorter distances and red longer. This means that dPGS remains close to its initial position near the KRRR group of amino acids in the case of the first generation dPGS. The second generation dPGS remains close to the RKRK site amino acids.

In the second step, we inspected all the MDs visually by preparing snapshots to check the structures. Snapshots revealed the flexible structure of the dPGS with its branches changing their orientation. To quantify its position, distances between the C3 atom of dPGS (located at the root of the three branches representing the center of mass of the dPGS, compare Figure 1a) and positively charged amino acids from the KRRR and RKRK sites have been calculated. These distances are presented visually in the heat maps which have been constructed as described in Section 4. Materials and Methods. Heat maps are presented in the Figure 5 for the 200-ns MD simulations of the dPGS (the first and the second generation) docked into the RKRK&KRRR box, and the other cases are given in the supporting information (Figures S4 and S5). Blue color in the maps represents short distances (less than 1 nm) between C3 atom of dPGS and positive amino acids in the RKRK and KRRR site. Distances longer than 1 nm are colored in shades of red. These heat maps allow separation of the MD simulations into two categories: (i) MD with stable position of the C3 atom next to the IL-6; (ii) MD where C3 atom changes position relative to RKRK and KRRR site. The five out of the six MDs belong to the first category where colors in the heat maps are mostly constant from 0 ns until 200 ns, meaning that the dPGS remains close to its initial position. These simulations include the first and the second generation dPGS docked into the KRRR, RKRK&KRRR sites and the second generation dPGS docked into the RKRK site. This is also visible in the snapshots where dPGS (colored red) does not change its position relative to RKRK (colored green) or KRRR amino acids (colored blue) (compare snapshots in the Figure 5 and Figure S5). The second category of the heat maps is represented in the remaining MD (the first generation dPGS docked into RKRK site) where dPGS moves from the RKRK to KRRR site. This is visible from the snapshots where dPGS changes its position (compare upper panel of the Figure S4a). Initial docking position (0 ns) has dPGS positioned clearly next to green amino acids from the RKRK site, while its position at 94 ns is close to the blue-colored amino acids from KRRR site. The heat map further allows to analyzes the MD results: the position of the dPGS shows that the shortest distance after 20 ns is between dPGS and R180 from the KRRR site, and the longest one is between dPGS and K42 from the RKRK site (compare heat map in the Figure S4a).

Additionally, the Molecular Mechanics Poisson–Boltzmann Surface Area (MM-PBSA) method [35,36] was used to calculate free energy of binding for IL-6 and different generations of the dPGS. This method determines binding energy by averaging over a large set of conformations from MD simulations. The calculated average binding energies from MD calculations are very similar for RKRK, KRRR and RKRKR&KRRR sites as shown in Table S1 (see Supporting information). These values are average of the two MDs obtained for IL6 bound to the dPGS of the first and the second generation. Altogether, the binding energy calculated using MM-PBSA does not contradict docking findings because the binding energies among sites are comparable.

Taken together, our results show that MD simulations confirm the docking results: (1) docking poses are stable and MD simulations preserve the complexes; (2) binding energies are similar for the RKRK and KRRR poses, which can be seen by the changing the position of the first generation dPGS (docked into RKRK site); (3) MD simulations qualitatively confirm previously described docking results.

## 3. Discussion

Our previous studies suggested that nanostructures with intrinsic anti-inflammatory activities can prevent or at least reduce deleterious effects of IL6 in rodents and in cell cultures. We also showed that dPGS binds directly to hIL6 [19,20]. However, the evidence for binding came from surface plasmon resonance (SPR) measurements with human recombinant IL-6 in vitro and the results provided binding constants but did not define the binding sites. The present study shows where dPGSs binds with the IL-6 and predicts the strength of non-covalent bonds.

In this study, the binding affinities provided in Table 1 and Table S1 are similar for both dPGS and heparin. The binding positions (Figure 2) and the Arpeggio analysis (Figure 4) reveal that these two ligands form ionic interaction with the residues R41 and R169 from the RKRK binding box. Additionally, polar interactions and hydrogen bonds formed in dPGS and heparin of the similar length include the same residues from hIL-6. Note that disaccharide heparin (with SO_3_^−^ group) with length of 13 Å corresponds to the dPGS of the first generation (assuming it is spherical in shape, it has 14 Å diameter) and the ionic interactions formed include R169, R180, R183, and R31 in both cases. Further, E173, Q176, and S177 are responsible for the polar interactions and hydrogen bonds. When comparing longer structures, pentasaccharide heparin and dPGS of the second generation have similar sizes (25 Å and 24 Å, respectively). Their interactions include R169, K172, and R31 for the formation of the ionic interactions while Q176, Q184, and S38 are involved in polar and hydrogen bonds. Due to its location in the center between the RKRK and KRRR sites, residue Q176 forms polar interactions and hydrogen bonds with almost all the ligands (except with the short disaccharide heparin). Analysis of the dPGS (Figure 3) shows presence of the hydrophobic interactions between dPGS and hIL-6, which are not present for heparin. These interactions originate mostly from the leucine residues L34 and L179. It is interesting to note that the pose obtained for the box (iii) RKRK&KRRR for the heparin disaccharide [OH] subunit is very similar to the one obtained in the RKRK box, but it has 0.2 kcal/mol stronger binding affinity. Interaction analysis shows that this small difference originates from the variation in the docking pose which is reflected in the scoring algorithm. This finding suggests that the binding affinities reported here are indeed very similar to those calculated for heparin, indicating that the dPGS can bind to similar positions with similar affinities as heparin, thus mimicking heparin interactions. In summary different approaches provide similar qualitative result that, in principle, heparin can be replaced by dPGS.

Our previously reported experimental data for dPGS-IL6 [20] and MOE by another group [37] are in line with our calculations. Our current studies are the first step in complex, multidisciplinary investigations of polyglycerol and other dendrimers interacting with proteins involved in pathological processes and under physiological conditions. Future quantitative binding assays with dPGS and analogs should provide experimental support of the theoretical findings presented here and complement our experimental data using SPR [20]. Considering the comparable strengths of binding for the two sites for heparin sulfate and dPGS it is likely that dPGS analyzed here will not provide highly protein specific binding because there are other proteins (such as fibronectin, laminin, apo E, etc. [24]) where similar amino acids yield comparable positively charged domains. Such interactions do not strongly depend on generation of dendrimers because a defined number of positive ions on the protein will dictate how many anionic sites can be involved in binding. Thus, any “bigger” dPGS structures e.g., the fifth generation of dPGS would not bind more or stronger than the second generation dPGS to IL-6. However, this does not exclude the possibility that other proteins different from hIL-6 will show remarkable dPGS generation-dependent effects. Considering Janus character of IL-6 i.e., its ability to exert both physiologically beneficial and detrimental effects, analogs of dPGS should be designed and tested in experiments with other proteins with positively charged domains. Such experiments should also include site mutation and deletion experiments. Another research direction could be to define if and how dPGS bind to receptors participating in trans-signaling [1]. Molecular modeling of the receptor sites could predict which modifications in dPGS structures would favor modulation of trans-signaling. Different IL-6 signaling modes are associated with different biological and clinical outcomes. Thus, dPGS analogs that could modulate classic versus trans-signaling could complement current armamentarium of therapeutics targeting IL-6 signaling. Antibody-based therapies to modulate IL-6 signaling do not discriminate between classic and trans- signaling except olam-kicept (sgp130Fc) which does not bind to IL-6 or IL-6R. Although several small molecules are in clinical trials for pathologies involving IL-6 signaling [1,38], a multidisciplinary approach to uncover intricate complexities in IL-6 signaling, could lead to dPGS analogs to complement and improve therapeutic interventions in inflammation. 

## 4. Materials and Methods

### 4.1. Docking Simulations

Docking simulations were carried out to predict the poses of the studied ligands (the first and the second generation of dendritic polyglycerol sulfate, and variable length heparins) on the two sites of the IL-6 (PDB entry 1IL6), and to estimate the binding affinity for the predicted poses. The molecular docking is used to predict the ligand structure at the receptor using computational modeling. Docking consists of two steps: sampling poses of the ligand at the binding site of the receptor; evaluating and sorting these poses using a scoring function, ideally ranking the experimental pose as the first one. Docking algorithms have two important segments: optimization algorithm and scoring function. Optimization algorithm identifies the rotatable bonds in the structures of the ligands and then fits the structure to the docking boxes while the scoring function evaluates the quasi-energy of the obtained receptor-ligand complex. In each optimization step, the binding score is evaluated until the lowest energy structure is found. Number of active torsions corresponds to the number of the bonds in ligand that can be rotated during the docking algorithm. Larger number of possible rotations makes it harder to find the binding position of the ligands [39]. Scoring function in the AutoDock Vina is a sum of intermolecular and intramolecular contributions. Free energy of the binding is evaluated from the intermolecular part and the Iterated Local Search global optimizer algorithm is used for optimization.

The activity of the proteins is based on their possibility to change their structure, and this is hard to model within docking studies. This is especially important when the active site of the protein includes flexible loops which have enormous number of degrees of freedom. As hIL-6 has possible binding sites for our ligands (positively charged residues) at the α-helices which do not undergo large structural changes, modeling using docking is reliable. Study on the heparin protein interactions reveals distinct role of water molecules [40] which can significantly contribute to protein-heparin interactions. Fruther, presence of the counterions in the solution influences the interaction of the dPGS and proteins [34]. In the present study, docking modeling did not include water molecules or ions because it exceeds the scope of the present work. Additional studies are needed to elucidate the role of the water and its influence on the process of heparin/dPGS binding to hIL-6. AutoDock Vina [41] software was used in this contribution. Preparation of the ligands for the docking was done using Chimera [42] Prep Dock Tool, which calculates charges for the ligand using AM1-BCC method (compare Figure S6). Docking was done with the default parameters, including energy_range = 3; exhaustiveness = 8, 9, 20 or 40; num_modes = 10. Structure of the dPGS was constructed and its energy minimized using Avogadro [43] software. Structure of the heparin was obtained from the ZINC15 data base (its reference code: ZINC53683651). The dockings were performed with flexible ligands to rigid hIL-6 with increasing exhaustiveness in each run. We defined and preformed docking with three different docking boxes: (i) box defined so that it includes amino acids Arg41, Lys42, Arg169 and Lys172 (RKRK site), (with the dimensions 22 × 30 × 25 Å3), centered at: (−7,6,15); (ii) the second box included amino acids Lys28, Arg31, Arg180 and Arg183 (KRRR site) with the same dimensions, but centered at (9,7,11); (iii) RKRK&KRRR box that included both amino acids from the (i) and (ii) with the dimensions 42 × 27 × 25 Å3 centered at (0,8,8). Because of the spatial proximity of the two possible binding sites we defined the third docking box so that it included both sites to check if one of the sites was preferred binding position, or if the elongated structures of heparin and dPGS can bind to both sites at the same time (Figure 1c).

The standalone version of Arpeggio, for calculating interatomic interactions classified in 15 different categories based on atom type, distance and angle terms [44], was used for the analysis of the interaction of each ligand with IL-6. Chimera software provided visual inspection and representation of the different complexes and plotting of the surfaces [42].

### 4.2. Details of the Molecular Dynamics Simulations

Gromacs version 2020.4 [45] was used to perform all-atom MD simulations of 6 different complexes in explicit solvent. We collected two 100 ns MD simulations for each complex of hIL-6 and dPGS obtained with docking (the first and the second generation of dPGS docked into the RKRK, KRRR, and RKRK&KRRR boxes). The Amber99sb force field [46] was used for hIL-6 in combination with the SPCE water model [47]. The General Amber Force Field (GAFF) [48,49] was used for dPGS, while the partial charges were calculated using the AM1-BCC quantum mechanical approach [50]. The simulations are preformed at 300 K and 1 bar, using the isothermal−isobaric (NPT) ensemble. We employed a dodecahedral box of water with a minimum distance between the edges of a box and protein atoms of 15 nm and applied periodic boundary conditions. Counterions (Na+ and Cl-) replaced solvent molecules to compensate for the protein net charge and make a system neutral. The systems were relaxed and equilibrated in a following way: (i) energy minimization by the steepest descent method (10,000 steps); (ii) solvent equilibration with restrained atomic positions of the protein and ligands using a harmonical potential (50 ps at 298 K); (iii) equilibration to the temperature of 298 K and pressure of 1 bar (100 ps each). Time-step was set on a 2 fs. The LINCS algorithm [51] was used to constrain heavyatom bonds, long-range electrostatic interactions were calculated using the ParticleMesh Ewald (PME) summation scheme [52] and van der Waals and short-range Coulomb interactions were cut at 0.9 nm. Built-in Gromacs tools were used for analysis of the simulations: *gmx mindist* (for calculations of the minimal distance between protein and its periodic image); *gmx pairdist* (for the distance analysis between dPGS and RKRK and KRRR site). Obtained distances for every step of the MD simulation were averaged in the 5-ns windows and plotted in the heat maps using Rstudio software [53]. Post-processing end-state method the Molecular Mechanics–Poisson Boltzmann Surface Area (MM-PBSA) was used to calculate free energies of binding for the IL6-dPGS complexes [35,36] as implemented in *g_mmpbsa* tool [54,55]. The free energy of the binding is calculated as difference between energies of the bound and unbound IL6 and ligands. It was averaged over two 100-ns MD simulations for each IL6-dPGS complex.

## Figures and Tables

**Table 1:** Summary of the binding affinities of the most favorable docking positions of ligands to hIL-6. For each ligand number of bonds that were rotated during the docking is stated.

Ligand/Docking Box	RKRK Site	KRRR Site	RKRK&KRRR
dPGS 1st generation 29 torsions	−5.7 kcal/mol	−6.0 kcal/mol	−6.0 kcal/mol
dPGS 2nd generation 53 torsions	−5.7 kcal/mol	−5.8 kcal/mol	−5.9 kcal/mol
Heparin disaccharide subunit [OH] 14 torsions	−6.3 kcal/mol	−6.6 kcal/mol	−6.5 kcal/mol
Heparin disaccharide subunit [OSO_3_^−^] 15 torsions	−5.9 kcal/mol	−6.0 kcal/mol	−5.7 kcal/mol
Heparin disaccharide subunit [OH] x2 28 torsions	−6.5 kcal/mol	−6.0 kcal/mol	−6.3 kcal/mol
Heparin disaccharide subunit [OSO_3_^−^] x2 30 torsions	−6.2 kcal/mol	−5.5 kcal/mol	−6.0 kcal/mol
Heparin pentasaccharide 37 torsions	/	/	−6.4 kcal/mol

## Supplementary Materials

Supplementary materials can be found at https://www.mdpi.com/1422-0067/22/5/2415/s1.

## Abbreviations

dPGS	Dendritic Polyglycerol Sulfates
hIL-6	Human Interleukin-6
GAGs	Glucosaminoglycans
MD	Molecular Dynamics
