# Supplementary material for: Insights into Interactions between Interleukin-6 and Dendritic Polyglycerols
Source: Int J Mol Sci. 2021 Feb 28;22(5):2415. doi: 10.3390/ijms22052415 (PMC7957513; doi:10.3390/ijms22052415)
Supplement: Supplementary file 1 [file ijms-22-02415-s001.pdf]

## Supporting information for:

# Insights into Interactions between Interleukin-6 and Dendritic Polyglycerols

**Željka Sanader Maršić [1,2,*], Dušica Maysinger [3,*], Vlasta Bonačić-Koutecký [2,4]**

[1] Faculty of Science, University of Split, Ruđera Boškovića 33, HR-21000 Split, Republic of Croatia; zsm@pmfst.hr

[2] Center of Excellence for Science and Technology-Integration of Mediterranean region (STIM) at Interdisciplinary Center for Advanced Sciences and Technology (ICAST), University of Split, Meštrovićevo šetalište 45, HR-21000 Split, Republic of Croatia; vbk@cms.hu-berlin.de

[3] McGill University, Department of Pharmacology & Therapeutics, McGill University, Montréal, QC H3G 1Y6, Canada; dusica.maysinger@mcgill.ca

[4] Department of Chemistry, Humboldt Universitat zu Berlin, Brook-Taylor-Strasse 2, 12489 Berlin, Germany; vbk@cms.hu-berlin.de

* Correspondence: zsm@pmfst.hr, dusica.maysinger@mcgill.ca

Fig. S1 presents NMR structure of heparin (pdb: 1HPN) illustrating its elongated structure with almost equally spaced charged sulfate groups. Distance between sulfate groups of the two subunits of heparin is highlighted.

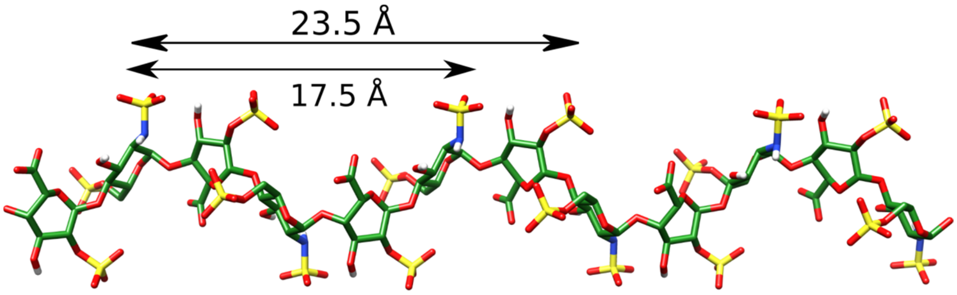


Fig. S2 shows the surface charge on the interleukin 6 (IL-6). Positively charged areas are colored blue, while negatively charged are red. The molecules in the stick representation are the most favorable docking poses for dPGS of the first and second generation. Single letter codes for the positive amino acids that defined RKRK and KRRR sites are indicated.

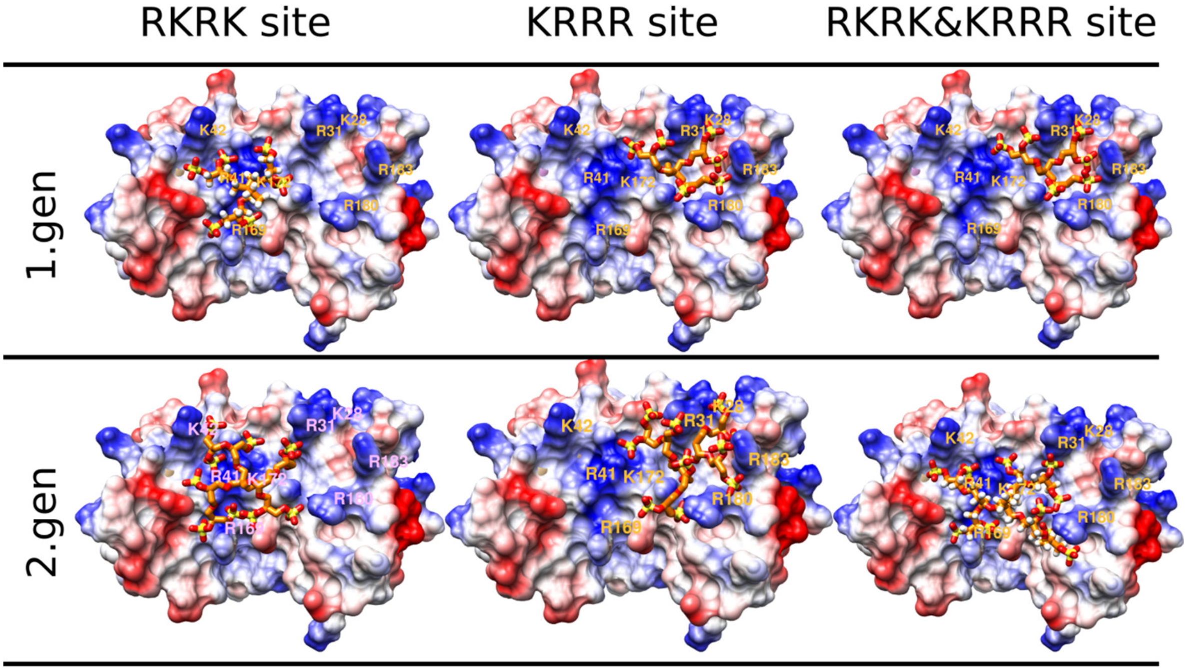

Fig. S3 presents the minimal distance between the protein and its periodic image for 12 MD simulations. Blue color presents the 200ns MD simulation, while the orange stands for 100ns replica. It is visible that the minimal distance is in all cases larger

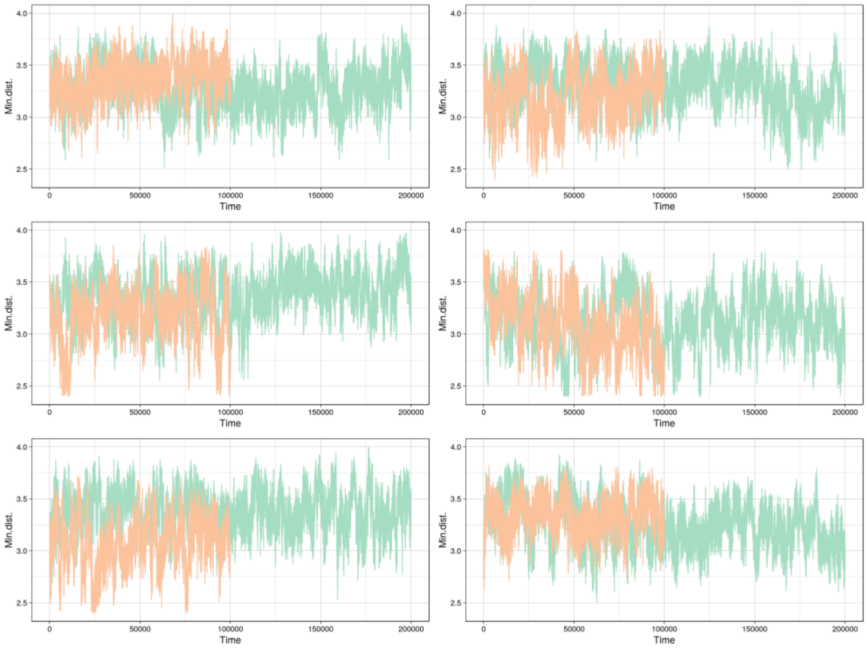

Fig. S4: Analysis of the MD simulations for 2 docked poses into RKRK box: a) dPGS of the first generation; and b) dPGS of the second generation. Upper parts show snapshots from the MD, with IL6 colored grey, dPGS colored red, amino acids from RKRK site are green and amino acids from KRRR skyblue. Heatmaps present analysis of the distances between central C3 atom (representing the "center of the mass") of the dPGS and positively charged aminoacids from RKRK (R41, K42, R169, K172) and KRRR (K28, R31, R180, R183) site during 200ns MD. Heatmap represents distances from 0.4 until 2.2 nm where blue color indicated shorter distances. In the case of the dPGS of the first generation, during the first 20ns dPGS is closer to amino acids from the RKRK site, and then it moves closer to the KRRR site (particulary to R180), while the second generation dPGS remains close to its initial docked position.

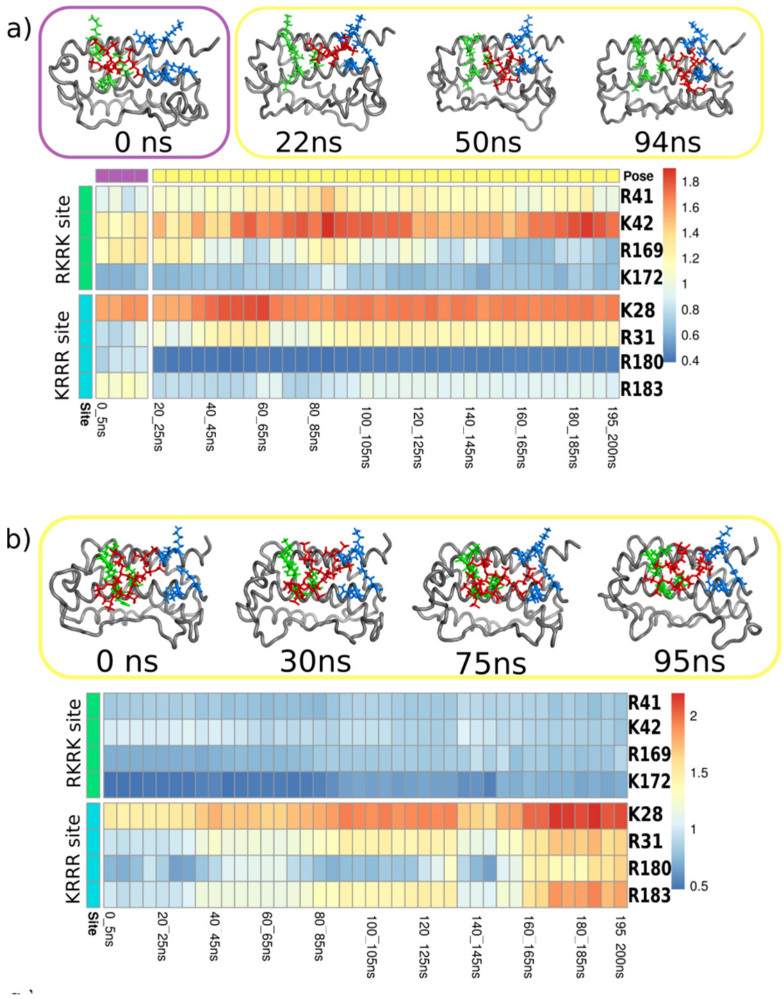

Fig S5: Analysis of the MD simulations for 2 docked poses into KRRR box: a) dPGS of the first generation; and b) dPGS of the second generation. Upper parts show snapshots from the MD, with IL6 colored grey, dPGS colored red, amino acids from RKRK site are green and amino acids from KRRR skyblue. Heatmaps present analysis of the distances between central C3 atom (representing the "center of the mass") of the dPGS and positively charged aminoacids from RKRK (R41, K42, R169, K172) and KRRR (K28, R31, R180, R183) site during 200ns MD. Heatmap represents distances from 0.4 until 2 nm where blue color indicated shorter distances which are stabile for the selected amino acids during the MDs showing that dPGS remains close to its initial position obtained by docking in the KRRR site in the case of the both generations of the dPGS.

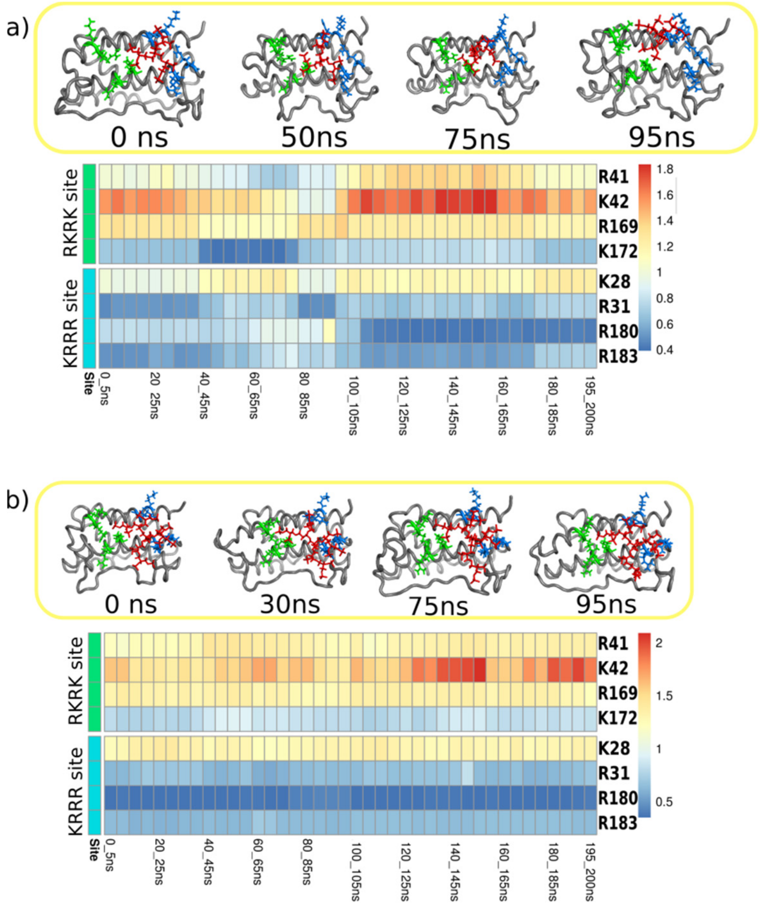

Fig. S6: a) Different types of the atoms of the dPGS used in the MD simulation are places at the nodes of the structure. One of the repeating branches is presented with the partial charges obtained with AM1-BCC method in the brackets. These charges were used in the docking and the MD simulations; b) partial charges at the atoms of heparin disaccharide subunit calculated using AM1-BCC method. Dashed bonds indicate difference between disaccharide subunits with and without sulfate group.

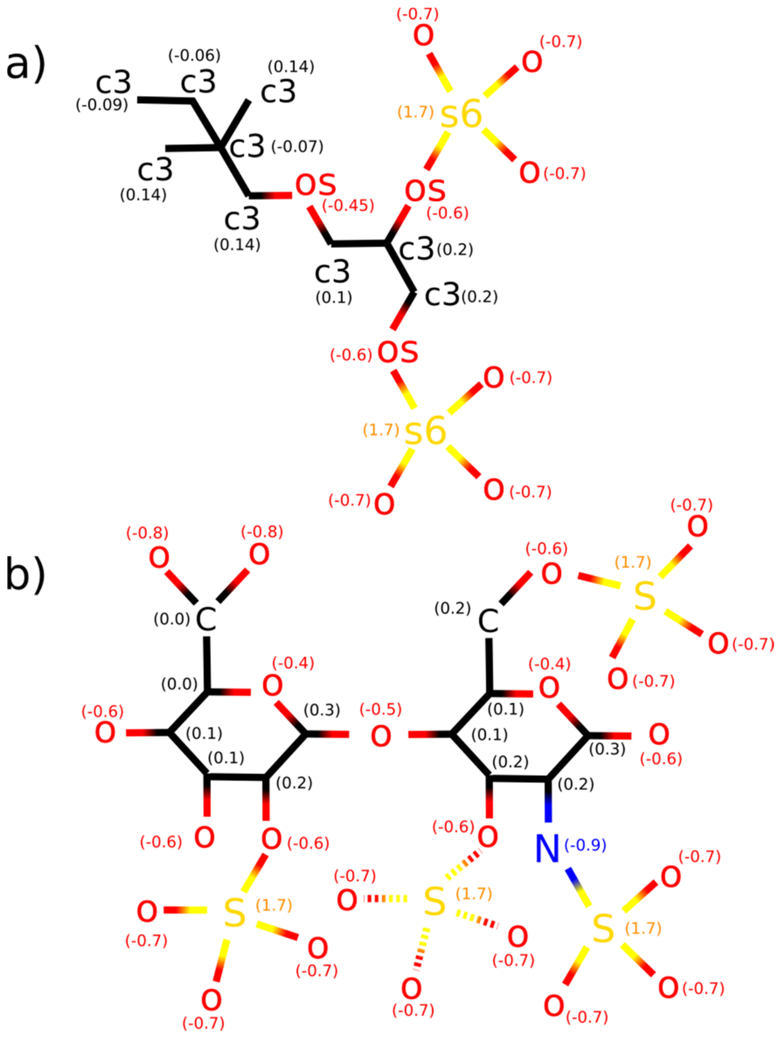

Table S1. Binding energy of the different generations of the dPGS to the IL-6 (expressed in kcal/mol) obtained using MMPBSA method. Results were averaged over two 100ns MD simulations.

| Ligand/<br>Docking box | RKRK | KRRR | RKRK&KRRR |
|---|---|---|---|
| dPGS<br>1st generation | -81.0 +/- 63.8 | -72.6+/-61.1 | -91.4+/-72.4 |
| dPGS<br>2nd generation | -113.3+/-62.9 | -200.0+/-91.1 | -113.6+/-87.7 |
